# Clinical and Pathological Roles of Ro/SSA Autoantibody System

**DOI:** 10.1155/2012/606195

**Published:** 2012-12-06

**Authors:** Ryusuke Yoshimi, Atsuhisa Ueda, Keiko Ozato, Yoshiaki Ishigatsubo

**Affiliations:** ^1^Department of Internal Medicine and Clinical Immunology, Yokohama City University Graduate School of Medicine, 3-9 Fukuura, Kanazawa-ku, Yokohama 236-0004, Japan; ^2^Program in Genomics of Differentiation, National Institute of Child Health and Human Development, National Institutes of Health, Bethesda, MD 20892, USA

## Abstract

Anti-Ro/SSA antibodies are among the most frequently detected autoantibodies against extractable nuclear antigens and have been associated with systemic lupus erythematosus (SLE) and Sjögren's syndrome (SS). Although the presence of these autoantibodies is one of the criteria for the diagnosis and classification of SS, they are also sometimes seen in other systemic autoimmune diseases. In the last few decades, the knowledge of the prevalence of anti-Ro/SSA antibodies in various autoimmune diseases and symptoms has been expanded, and the clinical importance of these antibodies is increasing. Nonetheless, the pathological role of the antibodies is still poorly understood. In this paper, we summarize the milestones of the anti-Ro/SSA autoantibody system and provide new insights into the association between the autoantibodies and the pathogenesis of autoimmune diseases.

## 1. Introduction

Systemic autoimmune diseases, including systemic lupus erythematosus (SLE) and Sjögren's syndrome (SS), are a category of medical conditions that affects multiple organs and are related to autoimmune responses. These are commonly characterized by the development of autoantibodies against intracellular autoantigens. In fact, diagnosis, classification, and prognosis often rely on specificity and levels of the autoantibodies, in addition to clinical symptoms and other laboratory evaluations. Among autoantigens, extractable nuclear antigens (ENA) are soluble cytoplasmic and nuclear components with over 100 different antigens described. The main antigens used in immunological laboratories for detection are Ro, La, Sm, RNP, Scl-70, and Jo1 [1]. Anti-Ro/SSA and anti-La/SSB antibodies are among the most frequently detected autoantibodies against ENA and have traditionally been associated with SLE, SS, subacute cutaneous lupus erythematosus (SCLE), and neonatal lupus erythematosus (NLE) [2–5]. Anti-Ro/SSA and anti-La/SSB can be detected in 70–100% and 40–90%, respectively, of patients with SS [6], and the presence of these autoantibodies is one of the criteria for the diagnosis and classification of SS [7, 8].

Anti-Ro/SSA and anti-La/SSB antibodies were originally described in 1961 as two precipitating antibodies reacting with antigens contained in extracts from salivary and lacrimal glands of patients with SS, termed SjD, and SjT, respectively [9]. SjD antigen was reported to be insensitive to trypsin or heat, while SjT antigen could be destroyed by the same treatment. In 1969, Clark et al. described the presence of antibodies in the sera of patients with SLE that reacted with ribonucleoprotein (RNP) antigens present in extracts of rabbit and human spleen [10]. The authors named the antibody “anti-Ro antibody” after the original patient in whom the antibodies were identified. The same group also found antibodies to another soluble cytoplasmic RNA protein antigen, “La” [11]. At about the same time, Alspaugh and Tan noted the existence of autoantibodies in the sera of many SS patients, which react with antigens termed “SSA” and “SSB,” [12]. SSB antigen was described also as “Ha”, an antigen targeted by sera from patients with SLE and SS [13]. Later, Ro and La were demonstrated to be antigenically identical to SSA and SSB [14]. 

While anti-Ro/SSA antibodies are primarily found in patients with SLE and SS, they are also sometimes seen in other systemic autoimmune diseases, such as systemic sclerosis (SSc), polymyositis/dermatomyositis (PM/DM), mixed connective tissue disease (MCTD), and rheumatoid arthritis (RA) [15, 16]. 

Although these anti-Ro/SSA antibodies have been used as a useful diagnostic marker for SLE and SS for decades, the pathological significance of the antibodies still remains to be clarified. In this paper, we summarize the milestones of the anti-Ro/SSA autoantibodies and provide new insights into the association between the autoantibodies and autoimmune diseases.

## 2. Two Autoantigens to SSA Autoantibodies, Ro52 and Ro60

In 1981, Lerner et al. showed that the Ro antigens associate with small cytoplasmic RNAs and form Ro-ribonucleoproteins (Ro-RNP) particles [45]. Later, Ro antigens were found to consist of two different proteins, Ro60 and Ro52. The target antigen for anti-Ro autoantibodies was first identified as a 60 kDa protein, which exists as RNP complexes with small cytoplasmic RNAs (hY-RNA) in 1984 [46]. Subsequently, complementary DNA (cDNA) of Ro60 was cloned [47, 48]. Ben-Chetrit et al. first discovered that a 52 kDa protein, named Ro52, was a part of the Ro antigens in 1988 [49], and three years later cDNA of human Ro52 was cloned [50, 51]. In humans, the *Ro60* gene is approximately 32 kb in size, located on chromosome 19, while the *Ro52* gene is 8.8 kb in size, located on chromosome 11. Although the Ro52 protein was initially suggested to be part of the Ro-hY-RNA complex with Ro60 [49, 52, 53], subsequent studies failed to confirm a direct interaction of the proteins [54, 55]. Recent studies provided evidence that Ro52 and Ro60 are localized to different cell compartments and that anti-Ro52 and anti-Ro60 antibodies have different clinical associations [15]. Thus it still remains unclear why autoantibodies to these two proteins are so closely linked.

Ro52 is an interferon (IFN)-inducible protein [56–64], and it is also induced by viral infection or Toll-like receptor (TLR) engagement via type I IFN induction [59, 62, 65, 66]. Following the first demonstration of Ro52 ubiquitin E3 ligase activity by Wada et al. several reports supporting the conclusion have been published by other groups [57, 67–70]. Recent studies, including *Ro52* gene disruption studies, suggest that Ro52 is a negative regulator for proinflammatory cytokine production. Yoshimi et al. noted an increase in production of NF-*κ*B-dependent cytokines, such as IL-1*β*, TNF*α*, and IL-6, that was observed in *Ro*52^−/−^ fibroblasts as compared to wild-type fibroblasts [62]. Data consistent with this report were published by another group [71, 72]. Another group reported that Ro52-deficient mice develop uncontrolled inflammation and systemic autoimmunity as a consequence of minor tissue injury caused by ear tagging [63]. In these mice, bone marrow-derived macrophages and splenocytes from the mutant mice released more inflammatory cytokines, IL-6, TNF*α*, type I IFN, and IL-23, upon TLR activation as compared to wild type. 

Several studies suggest possible associations between allelic polymorphisms of *Ro52* and the disease susceptibility and increased anti-Ro52 antibodies in SLE and SS [73–76]. Furthermore, about a twofold increase in the expression of Ro52 transcripts in peripheral blood mononuclear cells (PBMC) of patients with SLE and SS as compared with healthy controls has been reported [68]. 

On the other hand, Ro60 antigen binds to ~100 nt noncoding RNAs called hY-RNA [45–48, 77]. It was recently reported that the Ro60 protein, having a ring shape, binds to misfolded noncoding RNAs in vertebrate nuclei and acts as a quality checkpoint for RNA misfolding with molecular chaperones for defective RNAs [78, 79]. The misfolded RNAs are targeted by Ro60 for degradation [80–82]. The mice lacking the Ro60 protein develop an autoimmune syndrome characterized by autoantibody production, glomerulonephritis, and increased sensitivity to irradiation with ultraviolet light [83]. Thus, Ro60 may have a role to protect against autoimmune response.

## 3. Epitopes on Ro Autoantigens

Several studies showed that epitopes of Ro52 and Ro60 proteins have different conformational dependence [84–87]. On the Ro52 protein, most sera recognize linear epitopes in the denatured molecule, generally located in the leucine zipper site and not expressed on the surface of the native protein. In contrast, the epitopes recognized by anti-Ro60 antibodies are highly conformational and the antibodies largely lose the binding activity to the denatured protein. 

Dörner et al. showed that the central region, amino acid (aa) 153–245, is the main immunogenic region of Ro52 antigen, and the strongest antigenic epitopes are located within aa 197–245 region including the leucine zipper motif [88]. Antibody responses are directed against this major antigenic region regardless of the underlying autoimmune diseases, although the strikingly different levels of antibodies and the recognition of epitopes on aa 153–196 may be related to different disease expressions. Subsequently, sera from patients with SS were found to react heterogeneously to polyubiquitylated Ro52, probably due to their different antigenic epitopes [89]. 

McClain et al. described that the initial epitope on Ro60, prior to clinical disease onset, includes a peptide, aa 169–180 [90]. This epitope directly cross-reacts with a peptide, aa 58–72, of the Epstein-Barr virus nuclear antigen-1 (EBNA-1). The data support the hypothesis that the Epstein-Barr virus infection had a putative triggering effect by enhancing the development of autoantibodies to Ro60 through molecular mimicry [91, 92].

Polyclonal class-switched anti-Ro and anti-La responses can be elicited by immunization of normal mice with recombinant La protein [93]. In this process, the production of autoantibodies to different nonoverlapping regions of La was induced. Moreover, the same immunization rapidly induces the production of specific Ro60 antibodies. Reciprocally, mice immunized with Ro60 protein produced anti-La antibodies. This intra- and intermolecular spreading of autoantibody response suggests that the development of autoantibodies to multiple components of the Ro/La RNP complex may follow the initial response to a single epitope and suggest as a general explanation for the appearance of mixed autoantibody patterns in different systemic autoimmune diseases. 

The accessibility of Ro/La complex for the immune system is still unknown. Based on the antigen-driven immune response hypothesis, several works suggest that it could be related to an abnormal expression on the cell surface as a consequence of UV irradiation [94–96], oxidative stress [97], TNF*α* treatment [98], viral infection [99], or estradiol treatment [100]. Another mechanism for anti-Ro and anti-La antibodies' production could be related to antigen-containing apoptotic debris during programmed cell death [101, 102]. 

## 4. Anti-Ro Antibody and Demographic Feature

Anti-Ro antibodies can be detected alone in many sera while anti-La antibodies are usually accompanied by anti-Ro antibodies. HLA class II phenotype might support epitope spreading. The presence of anti-Ro and/or anti-La antibodies is more strikingly associated with HLA-DR3 and/or HLA-DR2 [103, 104]. HLA-DR3 is associated with both anti-Ro and anti-La antibody production while HLA-DR2 favors anti-SSA antibody synthesis [105]. HLA-DQ alleles are also linked to anti-Ro and anti-La antibody responses. Both DQ1 and DQ2 alleles are associated with high concentrations of these autoantibodies [106]. The data from restriction fragment length polymorphism (RFLP) analysis also indicated that HLA-DQ alleles are related to anti-Ro antibody response [107]. In this study, all patients with anti-Ro antibodies had a glutamine residue at position 34 of the outermost domain of the DQA1 chain and/or a leucine at position 26 of the outermost domain of the DQB1 chain. Patients with both anti-Ro and -La antibodies were more likely to have all four of their DQA1/DQB1 chains containing these amino acid residues than either anti-Ro-negative SLE patients or controls. These data implicate specific amino acid residues on both DQA1 and DQB1 chains located on the floor of the antigen-binding cleft of the HLA-DQA1:B1 heterodimer. 

Ro52 is an immunologically independent autoantibody system, and anti-Ro52 antibodies can exist without the presence of concomitant anti-Ro60 antibodies in systemic autoimmune diseases. Peene et al. found that anti-Ro52 antibodies are precipitin negative, not picked up by Ro enzyme-linked immunosorbent assays (ELISA) based on natural Ro proteins, and have no specific antinuclear antibody (ANA) fluorescence staining pattern [108]. As a consequence, anti-Ro52 antibodies are frequently not detected by classical Ro detection methods, which have a bias towards anti-Ro60 reactivity. Moreover, Schulte-Pelkum et al. showed that anti-Ro52 and anti-Ro60 reactivities can mask each other and that more than 20% of Ro positive sera can go undetected in assays that utilize blended antigens [15]. Therefore, the authors recommended that anti-Ro52 and anti-Ro60 antibodies should be tested separately.

There exists a paper showing that the prevalence of isolated anti-Ro52 antibodies was approximately 0.5%, and that detection did not lead to any significant clinical benefit as it was never the only explanation of symptoms [109]. On the other hand; several groups demonstrated the importance of separate detection of anti-Ro52 and anti-Ro60 antibodies when considering the diagnosis and, in particular, of patients with myositis [15, 41]. In a recent study, the frequency of anti-Ro52 antibodies was similar to that of anti-Ro60 in all groups but the myositis (35.4% versus 0.0%, *P* < 0.001) and SSc (19.0% versus 6.0%, *P* < 0.005) cohorts using the consensus of three different laboratory methods [15]. In the same study, the percentages of anti-Ro52 antibodies detected without anti-Ro60 antibodies also varied from 5.4% in childhood SLE to 35.4% in the myositis group. In the SS group, 63.2% of anti-Ro52 antibody-positive sera also had autoantibodies to Ro60.

Since Rutjes et al. found anti-Ro52 reactivity in 58% of anti-Jo-1 antibody-positive myositis sera in 1997 [41]; several groups confirmed the data in subsequent studies [15, 110–112]. The average coincidence of reactivity against Ro52 and Jo-1 was 70% (*P* = 0.0002, odds ratio = 14.17, *κ* = 0.54) in anti-Jo-1 antibody-positive sera of myositis patients in a recent study [15]. These observations also suggest previous conclusions that anti-Ro52 antibody is indeed an independent autoantibody for myositis [108]. 

Anti-Ro52 antibodies are frequently coexpressed with antibodies to soluble liver antigen (SLA) [40, 113]. The presence of anti-Ro52 antibodies has been reported in 77–96% of patients with anti-SLA antibodies, and patients with dual antibodies had a higher frequency of HLA DRB1*03 and lower occurrence of HLA DRB1*04 than patients with anti-Ro52 antibodies alone. 

## 5. Anti-Ro Antibodies and Autoimmune Diseases

Anti-Ro antibodies are the most prevalent autoantibodies among many autoimmune diseases, although their pathological role is still controversial [114]. Clinical manifestations related to anti-Ro antibodies are summarized in Table 1.

### 5.1. SLE and SS

Anti-Ro antibodies are frequently observed in association with SLE [115–117], SS/SLE overlap syndrome [118], SCLE [19], and NLE [25, 36, 119–122]. In contrast, anti-La antibody is more closely associated with SS. Anti-Ro antibodies can be detected in 70–100% and 40–90% of patients with SS and SLE, respectively, while anti-La antibodies can be detected in 35–70% and 45% of patients with SS and SLE, respectively [6]. SLE patients with C2 and C4 deficiency tend to have anti-Ro antibodies with cutaneous manifestations and polyarthritis, without renal or CNS features [116, 117, 123]. 

Anti-Ro and anti-La antibodies are found earlier than other SLE-related autoantibodies, such as anti-dsDNA, anti-ribonucleoprotein (RNP), and anti-Sm antibodies, and are present, on average, 3.4 years before the diagnosis of SLE [124]. Another paper also shows that the autoantibody type that appears first before the onset of symptoms is anti-Ro antibodies that appear at a mean of 6.6 years [125]. Some groups suggest a close association between anti-Ro antibodies and late onset SLE, with the onset of symptoms after the age of 50 [20, 126]. There are conflicting data as to the correlation of anti-Ro antibody titers with disease activity during the course of SLE and SS [127–131]. 

Anti-Ro antibodies have been reported to be associated with photosensitivity, SCLE, cutaneous vasculitis (palpable purpura), and hematological disorder (anemia, leukopenia, and thrombocytopenia) [17, 19, 21, 22, 44, 132]. Interstitial pneumonitis has been also closely associated with anti-Ro antibodies in patients with SLE, but there is so far no evidence of a direct involvement of the antibodies in the pathogenesis of the pulmonary disease [16, 20, 30, 31]. The relationship between anti-Ro antibodies and nonerosive deforming arthritis, called Jaccoud's arthropathy, has been reported [43, 133]. 

In SS, anti-Ro, and anti-La antibodies are present in the lacrimal fluid of some patients and their presence in serum or lacrimal fluid is associated with the severity of keratoconjunctivitis sicca [26]. High titers of anti-Ro and anti-La antibodies also have been shown to be associated with a greater incidence of extraglandular manifestations, especially purpura, leukopenia, and lymphopenia [23, 24].

### 5.2. NLE

NLE is a passively transferred autoimmune disease that occurs in some babies born to mothers with anti-Ro and/or anti-La antibodies [120, 134]. The most serious complication in the neonate is congenital complete heart block (CHB), which occurs in 1–5% of such pregnancies and 6–25% of subsequent pregnancies with a previously affected child with CHB [37]. 

Since the 1950s, it was recognized that maternal autoantibodies can cross the placenta and that fetuses of mothers with an autoimmune disease may develop isolated congenital complete atrioventricular block that has been already recognized as a distinct clinical entity [38, 135]. In the early 1980s, a close association between maternal anti-Ro and anti-La antibodies and CHB was shown [36, 136, 137]. 

Other features of NLE are frequently observed after birth and include cutaneous rash, hematological disorder (thrombocytopenia, leukopenia, and anemia), and liver dysfunction [25]. Unlike CHB, these symptoms of NLE usually resolve within 6 months after birth, coincident with the time of the clearance of the maternal antibodies from the infants' circulation. 

A recent paper described that all cardiac complications seen in neonates were associated with moderate to high (≥50 U/mL) maternal anti-Ro antibody levels, independently of anti-La antibody titers [38]. The event rate of CHB was 5% for prospectively screened fetuses with high anti-Ro antibody levels (≥50 U/mL; odds ratio: 7.8) and 0% for those exposed to lower titers. On the other hand, infants with prenatal exposure to high titers of anti-La antibodies (≥ 100 U/mL) were most likely to have noncardiac manifestations of NLE, with an event rate of 57% (odds ratio: 4.7). These findings suggest that the concentration of maternal autoantibodies, rather than their presence, is associated with the development of NLE. Thus fetal echocardiography should be reserved for women with high anti-Ro antibody titers [38]. 

As most mothers of neonates with NLE do not have any connective tissue disease, a previous suggestion that all pregnant women should be screened for anti-Ro antibodies irrespective of their symptoms or clinical status may be considered reasonable [138].

### 5.3. Other Autoimmune Diseases

Anti-Ro antibodies are found also in 3–11% of patients with SSc [15, 27, 32, 139] and associated with sicca symptoms and severe pulmonary involvement [27, 32, 33]. Anti-Ro antibodies are detectable in 5–15% of patients affected by idiopathic inflammatory myopathy, including polymyositis (PM) and dermatomyositis (DM). PM/DM patients with anti-Ro antibodies frequently showed a specific reactivity to Ro52 without Ro60 [15, 41, 42, 140]. The presence of anti-Ro52 antibodies is associated with anti-Jo-1 antibodies or other anti-aminoacyl transfer RNA synthetase (ARS) antibodies [15, 41, 140–142]. The coexistence of anti-Ro and anti-Jo-1 antibodies seems to be related to a more severe interstitial lung disease compared with the patients with anti-Jo-1 antibodies alone [34, 35]. 

Anti-Ro antibodies are detected in 3–15% of patients with RA[28, 29, 143]. Most RA patients with anti-Ro antibodies share the same extra-articular features, such as sicca, photosensitivity, purpura, leukopenia, anemia, and hypocomplementemia [18, 28, 29]. Some authors also have mentioned a strong association between anti-Ro antibodies and the development of side effects by treatment with gold salts or D-penicillamine [144–146]. In a recent report anti-Ro antibodies are suggested to be an independent factor associated with an insufficient response to tumor necrosis factor (TNF) inhibitors in patients with RA [147].

Anti-Ro52 antibodies have a high specificity in primary biliary cirrhosis (PBC), an autoimmune liver disease. They are found in 28% of patients with PBC and in a more advanced histological stage [39]. Higher serum bilirubin and IgM levels at the time of diagnosis are related to anti-Ro52 antibodies. Antibodies to Ro52 are also detected in 38% of patients with type 1 autoimmune hepatitis, and they, together with anti-SLA antibodies, are independently associated with the development of cirrhosis and hepatic death or liver transplantation [40].

## 6. Pathogenic Role of Anti-Ro Antibodies

Although the pathogenic role of autoantibodies in autoimmune disease has not yet been clarified, hypotheses have been put forward indicating that anti-Ro antibodies might have a direct role in damaging tissues. UV irradiation induces de novo synthesis of the Ro antigens in both the cytoplasm and the nucleus in keratinocytes [96]. Besides, UV irradiation increases the expression of the antigens on the cell surface [94, 96], enhancing the possibility of direct injury of keratinocytes by anti-Ro antibodies [95]. Based on the data, Norris developed a hypothetical model of the pathogenesis of photosensitivity [148]. (1) UV exposure leads to an increased synthesis and expression of Ro antigen on the surface of epidermal keratinocytes; (2) anti-Ro antibodies from the circulation bind to the antigens on the cell surface; (3) the Fc domains of the bound anti-Ro antibodies are recognized by lymphocytes, leading to keratinocyte death. This hypothesis was consistent with the following study showing that photosensitivity and titer of circulating anti-Ro/anti-La antibodies were directly correlated with the expression of Ro and La antigens in skin specimens of patients with SLE [149]. However, patients with SS and SLE with anti-Ro and/or anti-La antibodies only infrequently show photosensitivity [150].

Additional evidence for a direct pathogenic role of anti-Ro and anti-La can be found in studies of NLE. The cardiac damage is related to the expression of Ro and La antigens in fetal cardiac tissue from the 18th to 24th week, particularly located on the surface of cardiac myocytes [151–153]. Previous studies demonstrated that binding of anti-Ro and/or anti-La antibodies to apoptotic cardiocytes impairs their removal by healthy cardiocytes during the physiological cell deletion process in embryogenesis [154].It also increases urokinase plasminogen activator- (uPA-)/uPA receptor- (uPAR-) dependent plasminogen and TGF-*β* activation [155, 156]. Again, it is still unclear why NLE develops in only some but not all antibody-exposed fetuses. 

Interestingly, some anti-Ro antibody-positive adult patients with connective tissue disease may have disturbances in cardiac repolarization. Significantly increased mean corrected QT (QTc) intervals were present among anti-Ro antibody-positive patients when compared to anti-Ro antibody-negative individuals [157, 158]. The prolonged QTc interval seems to be directly attributable to the electrophysiological interference of anti-Ro antibodies with the inhibition of the I*κ*r current in cardiac myocytes [159]. Ventricular arrhythmias may also be more prevalent in those with anti-Ro antibodies [160]. 

Is there any possibility for anti-Ro antibodies to meet with Ro antigens inside the cell?Recently, it has been reported that IgG can enter the cytoplasm of nonimmune cells through the cell membrane together with virus [161]. In this paper, Ro52 acts as a cytosolic IgG receptor; it rapidly recruits incoming antibody-bound virus and targets it to the proteasomal degradation via its E3 ubiquitin ligase activity. This suggests the possibility of intracellular autoantigen-autoantibody interaction. A recent report shows that anti-Ro52 antibodies inhibit the E3 ligase activity of Ro52 by sterically blocking the E2/E3 interaction between Ro52 and UBE2E1 [162]. Although it still remains to be investigated whether enough anti-Ro52 autoantibodies can enter cells to sufficiently inhibit Ro52 function as a negative proinflammatory cytokine regulator, this inhibition may contribute to the pathogenesis of SLE and SS by inhibiting Ro52-mediated ubiquitylation.

## 7. Conclusions

Although anti-Ro antibodies have been used as a useful diagnostic marker for SLE and SS, they are the most prevalent autoantibodies among various autoimmune diseases. Above all, anti-Ro52 antibodies are specifically associated with myositis, SSc, and PBC. Furthermore, anti-Ro52 antibodies are related to a variety of symptoms in autoimmune diseases. Thus, separate measurement of anti-Ro52 and anti-Ro60 antibodies should be clinically useful. Anti-Ro52 antibodies may have pathological roles not only by damaging tissues directly but also by inhibiting the activity of Ro52 antigens. Further investigations into the Ro autoantigen-autoantibody system may offer a new strategy for treating autoimmune diseases.

## Figures and Tables

**Table 1:** Clinical manifestations related to anti-Ro antibodies.

Clinical manifestation	Disease	Ro52 specificity	Reference
Cutaneous manifestation			
Photosensitivity	SLE		[17]
	RA		[18]
Subacute cutaneous lesion	SLE/SCLE		[19, 20]
Purpura	SLE		[21, 22]
	SS		[23, 24]
	RA		[18]
Cutaneous NLE	NLE		[25]

Sicca symptom	SS		[26]
	SSc		[27]
	RA		[18, 28, 29]

Scleritis	RA		[28]

Interstitial lung disease	SLE	+	[16, 30, 31]
	SSc		[32, 33]
	PM/DM		[34, 35]

Congenital heart disease			
Complete heart block	NLE		[25, 36–38]
Prolonged QT interval	NLE		[25]

Liver dysfunction			
Liver function test abnormality	NLE		[25]
High serum bilirubin level	PBC	+	[39]
Advanced histological stage	PBC	+	[39]
	AIH-1	+	[40]

Musculoskeletal involvement			
Myositis	PM/DM	+	[15, 41, 42]
Arthritis	SLE		[43]

Hematological disorder			
Leukopenia	SS		[23]
	RA		[18]
Lymphopenia	SS		[23]
Neutropenia	SLE		[44]
	NLE		[25]
Anemia	NLE		[25]
	RA		[29]
Thrombocytopenia	NLE		[25]

Immunological disorder			
Hypocomplementemia	RA		[18]
High serum IgG level	SS		[23]
	RA		[28]
High serum IgM level	PBC	+	[39]

SLE: systemic lupus erythematosus; RA: rheumatoid arthritis; SCLE: subacute cutaneous lupus erythematosus; SS: Sjögren's syndrome; NLE: neonatal lupus erythematosus; SSc: systemic sclerosis; PM: polymyositis; DM: dermatomyositis; PBC: primary biliary cirrhosis; AIH-1: type 1 autoimmune hepatitis.

## References

[1] Prince HE, Hogrefe WR (1998). Evaluation of a line immunoblot assay for detection of antibodies recognizing extractable nuclear antigens. *Journal of Clinical Laboratory Analysis*.

[2] Chan EKL, Andrade LEC (1992). Antinuclear antibodies in Sjogren’s syndrome. *Rheumatic Disease Clinics of North America*.

[3] von Muhlen CA, Tan EM (1995). Autoantibodies in the diagnosis of systemic rheumatic diseases. *Seminars in Arthritis and Rheumatism*.

[4] Yamamoto K (2003). Pathogenesis of Sjogren's syndrome. *Autoimmunity Reviews*.

[5] Kobayashi R, Mii S, Nakano T, Harada H, Eto H (2009). Neonatal lupus erythematosus in Japan: a review of the literature. *Autoimmunity Reviews*.

[6] Wenzel J, Gerdsen R, Uerlich M, Bauer R, Bieber T, Boehm I (2001). Antibodies targeting extractable nuclear antigens: historical development and current knowledge. *British Journal of Dermatology*.

[7] Vitali C, Bombardieri S, Jonsson R (2002). Classification criteria for Sjögren’s syndrome: a revised version of the European criteria proposed by the American-European consensus group. *Annals of the Rheumatic Diseases*.

[8] Shiboski SC, Shiboski CH, Criswell L (2012). American College of Rheumatology classification criteria for Sjogren's syndrome: a data-driven, expert consensus approach in the Sjogren's International Collaborative Clinical Alliance cohort. *Arthritis Care and Research*.

[9] Anderson JR, Gray K, Beck JS, Kinnear WF (1961). Precipitating autoantibodies in Sjogren's disease. *The Lancet*.

[10] Clark G, Reichlin M, Tomasi TB (1969). Characterization of a soluble cytoplasmic antigen reactive with sera from patients with systemic lupus erythmatosus. *The Journal of Immunology*.

[11] Mattioli M, Reichlin M (1974). Heterogeneity of RNA protein antigens reactive with sera of patients with systemic lupus erythematosus. Description of a cytoplasmic nonribosomal antigen. *Arthritis and Rheumatism*.

[12] Alspaugh MA, Tan EM (1975). Antibodies to cellular antigens in Sjogren's syndrome. *The Journal of Clinical Investigation*.

[13] Akizuki M, Powers R, Holman HR (1977). A soluble acidic protein of the cell nucleus which reacts with serum from patients with systemic lupus erythematosus and Sjogren’s syndrome. *The Journal of Clinical Investigation*.

[14] Alspaugh M, Maddison P (1979). Resolution of the identity of certain antigen-antibody systems in systemic lupus erythematosus and Sjogren’s syndrome: an interlaboratory collaboration. *Arthritis and Rheumatism*.

[15] Schulte-Pelkum J, Fritzler M, Mahler M (2009). Latest update on the Ro/SS-a autoantibody system. *Autoimmunity Reviews*.

[16] Ghillani P, André C, Toly C (2011). Clinical significance of anti-Ro52 (TRIM21) antibodies non-associated with anti-SSA 60kDa antibodies: results of a multicentric study. *Autoimmunity Reviews*.

[17] Mond CB, Peterson MGE, Rothfield NF (1989). Correlation of anti-Ro antibody with photosensitivity rash in systemic lupus erythematosus patients. *Arthritis and Rheumatism*.

[18] Boire G, Menard HA, Gendron M, Lussier A, Myhal D (1993). Rheumatoid arthritis: anti-Ro antibodies define a non-HLA-DR4 associated clinicoserological cluster. *The Journal of Rheumatology*.

[19] McCauliffe DP (1997). Cutaneous diseases in adults associated with Anti-Ro/SS-A autoantibody production. *Lupus*.

[20] Hochberg MC, Boyd RE, Ahearn JM (1985). Systemic lupus erythematosus: a review of clinico-laboratory features and immunogenetic markers in 150 patients with emphasis on demographic subsets. *Medicine*.

[21] Fukuda MV, Lo SC, de Almeida CS, Shinjo SK (2009). Anti-Ro antibody and cutaneous vasculitis in systemic lupus erythematosus. *Clinical Rheumatology*.

[22] Alexander EL, Arnett FC, Provost TT, Stevens MB (1983). Sjogren’s syndrome: association of anti-Ro(SS-A) antibodies with vasculitis, hematologic abnormalities, and serologic hyperreactivity. *Annals of Internal Medicine*.

[23] Harley JB, Alexander EL, Bias WB (1986). Anti-Ro (SS-A) and anti-La (SS-B) in patients with Sjogren's syndrome. *Arthritis and Rheumatism*.

[24] Chung JK, Kim MK, Wee WR (2012). Prognostic factors for the clinical severity of keratoconjunctivitis sicca in patients with Sjogren's syndrome. *British Journal of Ophthalmology*.

[25] Cimaz R, Spence DL, Hornberger L, Silverman ED (2003). Incidence and spectrum of neonatal lupus erythematosus: a prospective study of infants born to mothers with anti-ro autoantibodies. *Journal of Pediatrics*.

[26] Toker E, Yavuz S, Direskeneli H (2004). Anti-Ro/SSA and anti-La/SSB autoantibodies in the tear fluid of patients with Sjogren's syndrome. *British Journal of Ophthalmology*.

[27] Drosos AA, Andonopoulos AP, Costopoulos JS, Stavropoulos ED, Papadimitriou CS, Moutsopoulos M (1988). Sjogren's syndrome in progressive systemic sclerosis. *The Journal of Rheumatology*.

[28] Cavazzana I, Franceschini F, Quinzanini M (2006). Anti-Ro/SSA antibodies in rheumatoid arthritis: clinical and immunologic associations. *Clinical and Experimental Rheumatology*.

[29] Skopouli FN, Andonopoulos AP, Moutsopoulos HM (1988). Clinical implications of the presence of anti-Ro(SSA) antibodies in patients with rheumatoid arthritis. *Journal of Autoimmunity*.

[30] Hedgpeth MT, Boulware DW (1988). Interstitial pneumonitis in antinuclear antibody-negative systemic lupus erythematosus: a new clinical manifestation and possible association with anti-Ro (SS-A) antibodies. *Arthritis and Rheumatism*.

[31] Provost TT, Watson R, Simmons-O’Brien E (1997). Anti-Ro(SS-A) antibody positive Sjogren’s/lupus erythematosus overlap syndrome. *Lupus*.

[32] Parodi A, Puiatti P, Rebora A (1991). Serological profiles as prognostic clues for progressive systemic scleroderma: the Italian experience. *Dermatologica*.

[33] Breit SN, Cairns D, Szentirmay A (1989). The presence of Sjogren’s syndrome is a major determinant of the pattern of interstitial lung disease in scleroderma and other connective tissue diseases. *The Journal of Rheumatology*.

[34] Vancsa A, Csipo I, Nemeth J, Devenyi K, Gergely L, Danko K (2009). Characteristics of interstitial lung disease in SS-A positive/Jo-1 positive inflammatory myopathy patients. *Rheumatology International*.

[35] La Corte R, Lo Mo Naco A, Locaputo A, Dolzani F, Trotta F (2006). In patients with antisynthetase syndrome the occurrence of anti-Ro/SSA antibodies causes a more severe interstitial lung disease. *Autoimmunity*.

[36] Buyon JP, Ben-Chetrit E, Karp S (1989). Acquired congenital heart block. Pattern of maternal antibody response to biochemically defined antigens of the SSA/Ro-SSB/La system in neonatal lupus. *The Journal of Clinical Investigation*.

[37] Brucato A, Frassi M, Franceschini F (1832). Risk of congenital complete heart block in newborns of mothers with anti-Ro/SSA antibodies detected by counterimmunoelectrophoresis: a prospective study of 100 women. *Arthritis and Rheumatism*.

[38] Jaeggi E, Laskin C, Hamilton R, Kingdom J, Silverman E (2010). The importance of the level of maternal Anti-Ro/SSA antibodies as a prognostic marker of the development of cardiac neonatal lupus erythematosus. A prospective study of 186 antibody-exposed fetuses and infants. *Journal of the American College of Cardiology*.

[39] Granito A, Muratori P, Muratori L (2007). Antibodies to SS-A/Ro-52kD and centromere in autoimmune liver disease: a clue to diagnosis and prognosis of primary biliary cirrhosis. *Alimentary Pharmacology and Therapeutics*.

[40] Montano-Loza AJ, Shums Z, Norman GL, Czaja AJ (2012). Prognostic implications of antibodies to Ro/SSA and soluble liver antigen in type 1 autoimmune hepatitis. *Liver International*.

[41] Rutjes SA, Vree Egberts WTM, Jongen P, Van Den Hoogen F, Pruijn GJM, Van Venrooij WJ (1997). Anti-Ro52 antibodies frequently co-occur with anti-Jo-1 antibodies in sera from patients with idiopathic inflammatory myopathy. *Clinical and Experimental Immunology*.

[42] Kubo M, Ihn H, Asano Y, Yamane K, Yazawa N, Tamaki K (2002). Prevalence of 52-kd and 60-kd Ro/SS-A autoantibodies in Japanese patients with polymyositis/dermatomyositis. *Journal of the American Academy of Dermatology*.

[43] Franceschini F, Cretti L, Quinzanini M, Rizzini FL, Cattaneo R (1994). Deforming arthropathy of the hands in systemic lupus erythematosus is associated with antibodies to SSA/Ro and to SSB/La. *Lupus*.

[44] Kurien BT, Newland J, Paczkowski C, Moore KL, Scofield RH (2000). Association of neutropenia in systemic lupus erythematosus (SLE) with anti-Ro and binding of an immunologically cross-reactive neutrophil membrane antigen. *Clinical and Experimental Immunology*.

[45] Lerner MR, Boyle JA, Hardin JA, Steitz JA (1981). Two novel classes of small ribonucleoproteins detected by antibodies associated with lupus erythematosus. *Science*.

[46] Wolin SL, Steitz JA (1984). The Ro small cytoplasmic ribonucleoproteins: Identification of the antigenic protein and its binding site on the Ro RNAs. *Proceedings of the National Academy of Sciences of the United States of America*.

[47] Ben-Chetrit E, Gandy BJ, Tan EM, Sullivan KF (1989). Isolation and characterization of a cDNA clone encoding the 60-kD component of the human SS-A/Ro ribonucleoprotein autoantigen. *The Journal of Clinical Investigation*.

[48] Deutscher SL, Harley JB, Keene JD (1988). Molecular analysis of the 60-kDa human Ro ribonucleoprotein. *Proceedings of the National Academy of Sciences of the United States of America*.

[49] Ben-Chetrit E, Chan EKL, Sullivan KF, Tan EM (1988). A 52-kD protein is a novel component of the SS-A/Ro antigenic particle. *Journal of Experimental Medicine*.

[50] Chan EKL, Hamel JC, Buyon JP, Tan EM (1991). Molecular definition and sequence motifs of the 52-kD component of human SS-A/Ro autoantigen. *The Journal of Clinical Investigation*.

[51] Itoh K, Itoh Y, Frank MB (1991). Protein heterogeneity in the human Ro/SSA ribonucleoproteins. The 52- and 60-kD Ro/SSA autoantigens are encoded by separate genes. *The Journal of Clinical Investigation*.

[52] Slobbe RL, Pluk W, Van Venrooij WJ, Pruijn GJM (1992). Ro ribonucleoprotein assembly in vitro identification of RNA-protein and protein-protein interactions. *Journal of Molecular Biology*.

[53] Kurien BT, Chambers TL, Thomas PY, Frank MB, Scofield RH (2001). Autoantibody to the leucine zipper region of 52 kDa Ro/SSA binds native 60 kDa Ro/SSA: identification of a tertiary epitope with components from 60 kDA Ro/SSA and 52 kDa Ro/SSA. *Scandinavian Journal of Immunology*.

[54] Boire G, Gendron M, Monast N, Bastin B, Menard HA (1995). Purification of antigenically intact Ro ribonucleoproteins; biochemical and immunological evidence that the 52-kD protein is not a Ro protein. *Clinical and Experimental Immunology*.

[55] Kelekar A, Saitta MR, Keene JD (1994). Molecular composition of Ro small ribonucleoprotein complexes in human cells. Intracellular localization of the 60- and 52-kD proteins. *The Journal of Clinical Investigation*.

[56] Rhodes DA, Ihrke G, Reinicke AT (2002). The 52 000 MW Ro/SS-A autoantigen in Sjögren’s syndrome/systemic lupus erythematosus (Ro52) is an interferon-*γ* inducible tripartite motif protein associated with membrane proximal structures. *Immunology*.

[57] Kong HJ, Anderson DE, Lee CH (2007). Cutting edge: autoantigen Ro52 is an interferon inducible E3 ligase that ubiquitinates IRF-8 and enhances cytokine expression in macrophages. *The Journal of Immunology*.

[58] Der SD, Zhou A, Williams BRG, Silverman RH (1998). Identification of genes differentially regulated by interferon *α*, *β*, or *γ* using oligonucleotide arrays. *Proceedings of the National Academy of Sciences of the United States of America*.

[59] Strandberg L, Ambrosi A, Espinosa A (2008). Interferon-*α* induces up-regulation and nuclear translocation of the Ro52 autoantigen as detected by a panel of novel Ro52-specific monoclonal antibodies. *Journal of Clinical Immunology*.

[60] Geiss GK, Salvatore M, Tumpey TM (2002). Cellular transcriptional profiling in influenza a virus-infected lung epithelial cells: the role of the nonstructural NS1 protein in the evasion of the host innate defense and its potential contribution to pandemic influenza. *Proceedings of the National Academy of Sciences of the United States of America*.

[61] Zimmerer JM, Lesinski GB, Radmacher MD, Ruppert A, Carson WE (2007). STAT1-dependent and STAT1-independent gene expression in murine immune cells following stimulation with interferon-alpha. *Cancer Immunology, Immunotherapy*.

[62] Yoshimi R, Chang TH, Wang H, Atsumi T, Morse HC, Ozato K (2009). Gene disruption study reveals a nonredundant role for TRIM21/Ro52 in NF-*κ*B-dependent cytokine expression in fibroblasts. *The Journal of Immunology*.

[63] Espinosa A, Dardalhon V, Brauner S (2009). Loss of the lupus autoantigen Ro52/Trim21 induces tissue inflammation and systemic autoimmunity by disregulating the IL-23-Th17 pathway. *Journal of Experimental Medicine*.

[64] Yoshimi R, Ishigatsubo Y, Ozato K (2012). Autoantigen TRIM21/Ro52 as a possible target for treatment of systemic lupus erythematosus. *International Journal of Rheumatology*.

[65] Rajsbaum R, Stoye JP, O’Garra A (2008). Type I interferon-dependent and -independent expression of tripartite motif proteins in immune cells. *European Journal of Immunology*.

[66] Ozato K, Shin DM, Chang TH, Morse HC (2008). TRIM family proteins and their emerging roles in innate immunity. *Nature Reviews Immunology*.

[67] Wada K, Kamitani T (2006). Autoantigen Ro52 is an E3 ubiquitin ligase. *Biochemical and Biophysical Research Communications*.

[68] Espinosa A, Zhou W, Ek M (2006). The Sjögren’s syndrome-associated autoantigen Ro52 is an E3 ligase that regulates proliferation and cell death. *The Journal of Immunology*.

[69] Sabile A, Meyer AM, Wirbelauer C (2006). Regulation of p27 degradation and S-phase progression by Ro52 RING finger protein. *Molecular and Cellular Biology*.

[70] Kim JY, Ozato K (2009). The sequestosome 1/p62 attenuates cytokine gene expression in activated macrophages by inhibiting IFN regulatory factor 8 and TNF receptor-associated factor 6/NF-*κ*B activity. *The Journal of Immunology*.

[71] Wada K, Niida M, Tanaka M, Kamitani T (2009). Ro52-mediated monoubiquitination of IKK*β* down-regulates NF-*κ*B signalling. *Journal of Biochemistry*.

[72] Niida M, Tanaka M, Kamitani T (2010). Downregulation of active IKK*β* by Ro52-mediated autophagy. *Molecular Immunology*.

[73] Frank MB, Itoh K, Fujisaku A, Pontarotti P, Mattei MG, Neas BR (1993). The mapping of the human 52-kD Ro/SSA autoantigen gene to human chromosome II, and its polymorphisms. *American Journal of Human Genetics*.

[74] Tsugu H, Horowitz R, Gibson N, Frank MB (1994). The location of a disease-associated polymorphism and genomic structure of the human 52-kDa Ro/SSA locus (SSA1). *Genomics*.

[75] Nakken B, Jonsson R, Bolstad AI (2001). Polymorphisms of the Ro52 gene associated with anti-Ro 52-kd autoantibodies in patients with primary Sjogren's syndrome. *Arthritis & Rheumatism*.

[76] Imanishi T, Morinobu A, Hayashi N (2005). A novel polymorphism of the SSA1 gene is associated with anti-SS-A/Ro52 autoantibody in Japanese patients with primary Sjögren’s syndrome. *Clinical and Experimental Rheumatology*.

[77] Hendrick JP, Wolin SL, Rinke J, Lerner MR, Steitz JA (1981). Ro small cytoplasmic ribonucleoproteins are a subclass of La ribonucleoproteins: further characterization of the Ro and La small ribonucleoproteins from uninfected mammalian cells. *Molecular and Cellular Biology*.

[78] Green CD, Long KS, Shi H, Wolin SL (1998). Binding of the 60-kDA Ro autoantigen to Y RNAs: evidence for recognition in the major groove of a conserved helix. *RNA*.

[79] Belisova A, Semrad K, Mayer O (2005). RNA chaperone activity of protein components of human Ro RNPs. *RNA*.

[80] Wolin SL, Reinisch KM (2006). The Ro 60 kDa autoantigen comes into focus: Interpreting epitope mapping experiments on the basis of structure. *Autoimmunity Reviews*.

[81] O’Brien CA, Wolin SL (1994). A possible role for the 60-kD Ro autoantigen in a discard pathway for defective 5S rRNA precursors. *Genes and Development*.

[82] Chen X, Smith JD, Shi H, Yang DD, Flavell RA, Wolin SL (2003). The Ro autoantigen binds misfolded U2 small nuclear RNAs and assists mammalian cell survival after UV irradiation. *Current Biology*.

[83] Xue D, Shi H, Smith JD (2003). A lupus-like syndrome develops in mice lacking the Ro 60-kDa protein, a major lupus autoantigen. *Proceedings of the National Academy of Sciences of the United States of America*.

[84] Itoh Y, Reichlin M (1992). Autoantibodies to the Ro/SSA antigen are conformation dependent. I: anti-60 kD antibodies are mainly directed to the native protein; anti-52 kD antibodies are mainly directed to the denatured protein. *Autoimmunity*.

[85] Boire G, Craft J (1989). Biochemical and immunological heterogeneity of the Ro ribonucleoprotein particles. Analysis with sera specific for the Ro(hY5) particle. *The Journal of Clinical Investigation*.

[86] Boire G, Lopez-Longo FJ, Lapointe S, Menard HA (1991). Sera from patients with autoimmune disease recognize conformational determinants on the 60-kd Ro/SS-A protein. *Arthritis and Rheumatism*.

[87] Itoh Y, Itoh K, Frank MB, Reichlin M (1993). Autoantibodies to the Ro/SSA autoantigen are conformation dependent II: antibodies to the denatured form of 52 kD Ro/SSA are a cross reacting subset of antibodies to the native 60 kD Ro/SSA molecule. *Autoimmunity*.

[88] Dörner T, Feist E, Wagenmann A (1996). Anti-52 kDa Ro(SSA) autoantibodies in different autoimmune diseases preferentially recognize epitopes on the central region of the antigen. *The Journal of Rheumatology*.

[89] Fukuda-Kamitani T, Kamitani T (2002). Ubiquitination of Ro52 autoantigen. *Biochemical and Biophysical Research Communications*.

[90] McClain MT, Heinlen LD, Dennis GJ, Roebuck J, Harley JB, James JA (2005). Early events in lupus humoral autoimmunity suggest initiation through molecular mimicry. *Nature Medicine*.

[91] Doria A, Canova M, Tonon M (2008). Infections as triggers and complications of systemic lupus erythematosus. *Autoimmunity Reviews*.

[92] Poole BD, Templeton AK, Guthridge JM, Brown EJ, Harley JB, James JA (2009). Aberrant epstein-barr viral infection in systemic lupus erythematosus. *Autoimmunity Reviews*.

[93] Topfer F, Gordon T, Mccluskey J (1995). Intra- and intermolecular spreading of autoimmunity involving the nuclear self-antigens La (SS-B) and Ro (SS-A). *Proceedings of the National Academy of Sciences of the United States of America*.

[94] Furukawa F, Kashihara-Sawami M, Lyons MB, Norris DA (1990). Binding of antibodies to the extractable nuclear antigens SS-A/Ro and SS-B/La is induced on the surface of human keratinocytes by ultraviolet light (UVL): implications for the pathogenesis of photosensitive cutaneous lupus. *Journal of Investigative Dermatology*.

[95] Golan TD, Elkon KB, Gharavi AE, Krueger JG (1992). Enhanced membrane binding of autoantibodies to cultured keratinocytes of systemic lupus erythematosus patients after ultraviolet B/ultraviolet a irradiation. *The Journal of Clinical Investigation*.

[96] LeFeber WP, Norris DA, Ryan SR (1984). Ultraviolet light induces binding of antibodies to selected nuclear antigens on cultured human keratinocytes. *The Journal of Clinical Investigation*.

[97] Saegusa J, Kawano S, Koshiba M (2002). Oxidative stress mediates cell surface expression of SS-A/Ro antigen on keratinocytes. *Free Radical Biology and Medicine*.

[98] Dorner T, Hucko M, Mayet WJ, Trefzer U, Burmester GR, Hiepe F (1995). Enhanced membrane expression of the 52 kDa Ro(SS-A) and La(SS-B) antigens by human keratinocytes induced by TNF*α*. *Annals of the Rheumatic Diseases*.

[99] Baboonian C, Venables PJW, Booth J, Williams DG, Roffe LM, Maini RN (1989). Virus infection induces redistribution and membrane localization of the nuclear antigen La (SS-B): a possible mechanism for autoimmunity. *Clinical and Experimental Immunology*.

[100] Furukawa F, Lyons MB, Lee LA, Coulter SN, Norris DA (1988). Estradiol enhances binding to cultured human keratinocytes of antibodies specific for SS-A/Ro and SS-B/La. Another possible mechanism for estradiol influence of lupus erythematosus. *The Journal of Immunology*.

[101] Casciola-Rosen LA, Anhalt G, Rosen A (1994). Autoantigens targeted in systemic lupus erythematosus are clustered in two populations of surface structures on apoptotic keratinocytes. *Journal of Experimental Medicine*.

[102] Routsias JG, Tzioufas AG (2010). Autoimmune response and target autoantigens in Sjogren’s syndrome. *European Journal of Clinical Investigation*.

[103] Wilson RW, Provost TT, Bias WB (1984). Sjogren’s syndrome. Influence of multiple HLA-D region alloantigens on clinical and serologic expression. *Arthritis and Rheumatism*.

[104] Hamilton RG, Harley JB, Bias WB (1988). Two Ro (SS-A) autoantibody responses in systemic lupus erythematosus: correlation of HLA-DR/DQ specificities with quantitative expression of Ro (SS-A) autoantibody. *Arthritis and Rheumatism*.

[105] Gottenberg JE, Busson M, Loiseau P (2003). In primary Sjögren’s syndrome, HLA class II is associated exclusively with autoantibody production and spreading of the autoimmune response. *Arthritis and Rheumatism*.

[106] Harley JB, Reichlin M, Arnett FC (1986). Gene interaction at HLA-DQ enhances autoantibody production in primary Sjogren’s syndrome. *Science*.

[107] Reveille JD, Macleod MJ, Whittington K, Arnett FC (1991). Specific amino acid residues in the second hypervariable region of HLA-DQA1 and DQB1 chain genes promote the Ro (SS-A)/La (SS-B) autoantibody responses. *The Journal of Immunology*.

[108] Peene I, Meheus L, De Keyser S, Humbel R, Veys EM, De Keyser F (2002). Anti-Ro52 reactivity is an independent and additional serum marker in connective tissue disease. *Annals of the Rheumatic Diseases*.

[109] Langguth DM, Morris S, Clifford L (2007). Specific testing for “isolated” anti-52 kDa SSA/Ro antibodies during standard anti-extractable nuclear antigen testing is of limited clinical value. *Journal of Clinical Pathology*.

[110] Rozman B, Bozic B, Kos-Golja M, Plesivcnik-Novljan M, Kveder T (2000). Immunoserological aspects of idiopathic inflammatory muscle disease. *Wiener Klinische Wochenschrift*.

[111] Brouwer R, Hengstman GJD, Vree Egberts W (2001). Autoantibody profiles in the sera of European patients with myositis. *Annals of the Rheumatic Diseases*.

[112] Koenig M, Fritzler MJ, Targoff IN, Troyanov Y, Senécal JL (2007). Heterogeneity of autoantibodies in 100 patients with autoimmune myositis: insights into clinical features and outcomes. *Arthritis Research and Therapy*.

[113] Eyraud V, Chazouilleres O, Ballot E, Corpechot C, Poupon R, Johanet C (2009). Significance of antibodies to soluble liver antigen/liver pancreas: a large French study. *Liver International*.

[114] Franceschini F, Cavazzana I (2005). Anti-Ro/SSA and La/SSB antibodies. *Autoimmunity*.

[115] Maddison PJ, Provost TT, Reichlin M (1981). Serological findings in patients with ’ANA-negative’ systemic lupus erythematosus. *Medicine*.

[116] Provost TT, Arnett FC, Reichlin M (1983). Homozygous C2 deficiency, lupus erythematosus, and anti-Ro (SSA) antibodies. *Arthritis and Rheumatism*.

[117] Tappeiner G, Hintner H, Scholz S (1982). Systemic lupus erythematosus in hereditary deficiency of the fourth component of complement. *Journal of the American Academy of Dermatology*.

[118] Provost TT, Talal N, Harley JB, Reichlin M, Alexander E (1988). The relationship between anti-Ro (SS-A) antibody-positive Sjogren’s syndrome and anti-Ro (SS-A) antibody-positive lupus erythematosus. *Archives of Dermatology*.

[119] Buyon JP, Winchester RJ, Slade SG (1993). Identification of mothers at risk for congenital heart block and other neonatal lupus syndromes in their children: comparison of enzyme-linked immunosorbent assay and immunoblot for measurement of anti-SS-A/Ro and anti- SS-B/La antibodies. *Arthritis and Rheumatism*.

[120] Buyon JP, Clancy RM, Friedman DM (2009). Cardiac manifestations of neonatal lupus erythematosus: guidelines to management, integrating clues from the bench and bedside. *Nature Clinical Practice Rheumatology*.

[121] Lockshin MD, Bonfa E, Elkon K, Druzin ML (1988). Neonatal lupus risk to newborns of mothers with systemic lupus erythematosus. *Arthritis and Rheumatism*.

[122] Garcia S, Nascimento JHM, Bonfa E (1994). Cellular mechanism of the conduction abnormalities induced by serum from anti-Ro/SSA-positive patients in rabbit hearts. *The Journal of Clinical Investigation*.

[123] Meyer O, Hauptmann G, Tappeiner G (1985). Genetic deficiency of C4, C2 or C1q and lupus syndromes. Association with anti-Ro (SS-A) antibodies. *Clinical and Experimental Immunology*.

[124] Arbuckle MR, McClain MT, Rubertone MV (2003). Development of autoantibodies before the clinical onset of systemic lupus erythematosus. *The New England Journal of Medicine*.

[125] Eriksson C, Kokkonen H, Johansson M, Hallmans G, Wadell G, Rantapää-Dahlqvist S (2011). Autoantibodies predate the onset of systemic lupus erythematosus in northern Sweden. *Arthritis Research and Therapy*.

[126] Catoggio LJ, Skinner RP, Smith G, Maddison PJ (1984). Systemic lupus erythematosus in the elderly: clinical and serological characteristics. *The Journal of Rheumatology*.

[127] Praprotnik S, Bozic B, Kveder T, Rozman B (1999). Fluctuation of anti-Ro/SS-A antibody levels in patients with systemic lupus erythematosus and Sjogren’s syndrome: a prospective study. *Clinical and Experimental Rheumatology*.

[128] Scopelitis E, Biundo JJ, Alspaugh MA (1980). Anti-SS-A antibody and other antinuclear antibodies in systemic lupus erythematosus. *Arthritis and Rheumatism*.

[129] Derksen RHWM, Meilof JF (1992). Anti-Ro/SS-A and anti-La/SS-B autoantibody levels in relation to systemic lupus erythematosus disease activity and congenital heart block: a longitudinal study comprising two consecutive pregnancies in a patient with systemic lupus erythematosus. *Arthritis and Rheumatism*.

[130] Wahren M, Tengnér P, Gunnarsson I (1998). Ro/SS-A and La/SS-B antibody level variation in patients with Sjogren’s syndrome and systemic lupus erythematosus. *Journal of Autoimmunity*.

[131] Hassan AB, Lundberg IE, Isenberg D, Wahren-Herlenius M (2002). Serial analysis of Ro/SSA and La/SSB antibody levels and correlation with clinical disease activity in patients with systemic lupus erythematosus. *Scandinavian Journal of Rheumatology*.

[132] Maddison P, Mogavero H, Provost TT, Reichlin M (1979). The clinical significance of autoantibodies to a soluble cytoplasmic antigen in systemic lupus erythematosus and other connective tissue diseases. *The Journal of Rheumatology*.

[133] Simmons-O’Brien E, Chen S, Watson R (1995). One hundred anti-Ro (SS-A) antibody positive patients: a 10-year follow-up. *Medicine*.

[134] Brucato A, Cimaz R, Caporali R, Ramoni V, Buyon J (2011). Pregnancy outcomes in patients with autoimmune diseases and anti-Ro/SSA antibodies. *Clinical Reviews in Allergy and Immunology*.

[135] Chameides L, Truex RC, Vetter V, Rashkind WJ, Galioto FM, Noonan JA (1977). Association of maternal systemic lupus erythematosus with congenital complete heart block. *The New England Journal of Medicine*.

[136] Weston WL, Harmon C, Peebles C (1982). A serological marker for neonatal lupus erythematosus. *British Journal of Dermatology*.

[137] Lee LA, Bias WB, Arnett FC (1983). Immunogenetics of the neonatal lupus syndrome. *Annals of Internal Medicine*.

[138] Claus R, Hickstein H, Külz T (2006). Identification and management of fetuses at risk for, or affected by, congenital heart block associated with autoantibodies to SSA (Ro), SSB (La), or an HsEg5-like autoantigen. *Rheumatology International*.

[139] Fujimoto M, Shimozuma M, Yazawa N (1997). Prevalence and clinical relevance of 52-kDa and 60-kDa Ro/SS-A autoantibodies in Japanese patients with systemic sclerosis. *Annals of the Rheumatic Diseases*.

[140] Frank MB, McCubbin V, Trieu E, Wu Y, Isenberg DA, Targoff IN (1999). The association of anti-Ro52 autoantibodies with myositis and scleroderma autoantibodies. *Journal of Autoimmunity*.

[141] Targoff IN, Miller FW, Medsger TA, Oddis CV (1997). Classification criteria for the idiopathic inflammatory myopathies. *Current Opinion in Rheumatology*.

[142] Kao AH, Lacomis D, Lucas M, Fertig N, Oddis CV (2004). Anti-signal recognition particle autoantibody in patients with and patients without idiopathic inflammatory myopathy. *Arthritis and Rheumatism*.

[143] Schneeberger E, Citera G, Heredia M, Cocco JM (2008). Clinical significance of anti-Ro antibodies in rheumatoid arthritis. *Clinical Rheumatology*.

[144] Moutsopoulos HM, Giotaki H, Maddison PJ, Mavridis AC, Drosos AA, Skopouli FN (1984). Antibodies to cellular antigens in Greek patients with autoimmune rheumatic diseases: anti-Ro (SSA) antibody a possible marker of penicillamine-D intolerance. *Annals of the Rheumatic Diseases*.

[145] Moutsopoulos HM, Skopouli FN, Sarras AK (1985). Anti-Ro(SSA) positive rheumatoid arthritis (RA): a clinicoserological group of patients with high incidence of D-penicillamine side effects. *Annals of the Rheumatic Diseases*.

[146] Tishler M, Golbrut B, Shoenfeld Y, Yaron M (1994). Anti-Ro(SSA) antibodies in patients with rheumatoid arthritis a possible marker for gold induced side effects. *The Journal of Rheumatology*.

[147] Matsudaira R, Tamura N, Sekiya F, Ogasawara M, Yamanaka K, Takasaki Y (2011). Anti-Ro/SSA antibodies are an independent factor associated with an insufficient response to tumor necrosis factor inhibitors in patients with rheumatoid arthritis. *The Journal of Rheumatology*.

[148] Norris DA (1993). Pathomechanisms of photosensitive lupus erythematosus. *Journal of Investigative Dermatology*.

[149] Ioannides D, Golden BD, Buyon JP, Bystryn JC (2000). Expression of SS-A/Ro and SS-B/La antigens in skin biopsy specimens of patients with photosensitive forms of lupus erythematosus. *Archives of Dermatology*.

[150] Sontheimer RD (1996). Photoimmunology of lupus erythematosus and dermatomyositis: a speculative review. *Photochemistry and Photobiology*.

[151] Deng JS, Bair LW, Shen-Schwarz S, Ramsey-Goldman R, Medsger T (1987). Localization of Ro (SS-A) antigen in the cardiac conduction system. *Arthritis and Rheumatism*.

[152] Taylor PV, Scott JS, Gerlis LM, Esscher E, Scott O (1986). Maternal antibodies against fetal cardiac antigens in congenital complete heart block. *The New England Journal of Medicine*.

[153] Tseng CE, Buyon JP (1997). Neonatal lupus syndromes. *Rheumatic Disease Clinics of North America*.

[154] Clancy RM, Neufing PJ, Zheng P (2006). Impaired clearance of apoptotic cardiocytes is linked to anti-SSA/Ro and -SSB/La antibodies in the pathogenesis of congenital heart block. *The Journal of Clinical Investigation*.

[155] Briassouli P, Komissarova EV, Clancy RM, Buyon JP (2010). Role of the urokinase plasminogen activator receptor in mediating impaired efferocytosis of anti-SSA/Ro-bound apoptotic cardiocytes implications in the pathogenesis of congenital heart block. *Circulation Research*.

[156] Briassouli P, Rifkin D, Clancy RM, Buyon JP (2011). Binding of anti-SSA antibodies to apoptotic fetal cardiocytes stimulates urokinase plasminogen activator (uPA)/uPA receptor-dependent activation of TGF-beta and potentiates fibrosis. *The Journal of Immunology*.

[157] Lazzerini PE, Acampa M, Guideri F (2004). Prolongation of the corrected QT interval in adult patients with Anti-Ro/SSA-positive connective tissue diseases. *Arthritis and Rheumatism*.

[158] Bourre-Tessier J, Clarke AE, Huynh T (2011). Prolonged corrected QT interval in anti-Ro/SSA-positive adults with systemic lupus erythematosus. *Arthritis Care and Research*.

[159] Lazzerini PE, Capecchi PL, Acampa M (2009). Arrhythmogenic effects of anti-Ro/SSA antibodies on the adult heart: more than expected?. *Autoimmunity Reviews*.

[160] Lazzerini PE, Capecchi PL, Guideri F (2007). Comparison of frequency of complex ventricular arrhythmias in patients with positive versus negative anti-Ro/SSA and connective tissue disease. *American Journal of Cardiology*.

[161] Mallery DL, McEwan WA, Bidgood SR, Towers GJ, Johnson CM, James LC (2010). Antibodies mediate intracellular immunity through tripartite motif-containing 21 (TRIM21). *Proceedings of the National Academy of Sciences of the United States of America*.

[162] Espinosa A, Hennig J, Ambrosi A (2011). Anti-Ro52 autoantibodies from patients with Sjogren's syndrome inhibit the Ro52 E3 ligase activity by blocking the E3/E2 interface. *The Journal of Biological Chemistry*.

